# Fine Particulate Matter Constituents Associated with Cardiovascular Hospitalizations and Mortality in New York City

**DOI:** 10.1289/ehp.1002667

**Published:** 2010-12-17

**Authors:** Kazuhiko Ito, Robert Mathes, Zev Ross, Arthur Nádas, George Thurston, Thomas Matte

**Affiliations:** 1 New York University School of Medicine, Tuxedo, New York, USA; 2 New York City Department of Health and Mental Hygiene, New York, New York, USA; 3 ZevRoss Spatial Analysis, Ithaca, New York, USA; 4 Hunter College, City University of New York School of Public Health, New York, New York, USA

**Keywords:** air pollution, chemical species, New York City, particulate matter, traffic

## Abstract

**Background:**

Recent time-series studies have indicated that both cardiovascular disease (CVD)mortality and hospitalizations are associated with particulate matter (PM). However, seasonal patterns of PM associations with these outcomes are not consistent, and PM components responsible for these associations have not been determined. We investigated this issue in New York City (NYC), where PM originates from regional and local combustion sources.

**Objective:**

In this study, we examined the role of particulate matter with aerodynamic diameter ≤ 2.5 μm (PM_2.5_) and its key chemical components on both CVD hospitalizations and on mortality in NYC.

**Methods:**

We analyzed daily deaths and emergency hospitalizations for CVDs among persons ≥ 40 years of age for associations with PM_2.5_, its chemical components, nitrogen dioxide (NO_2_), carbon monoxide, and sulfur dioxide for the years 2000–2006 using a Poisson time-series model adjusting for temporal and seasonal trends, temperature effects, and day of the week. We estimated excess risks per interquartile-range increases at lags 0 through 3 days for warm (April through September) and cold (October through March) seasons.

**Results:**

The CVD mortality series exhibit strong seasonal trends, whereas the CVD hospitalization series show a strong day-of-week pattern. These outcome series were not correlated with each other but were individually associated with a number of PM_2.5_ chemical components from regional and local sources, each with different seasonal patterns and lags. Coal-combustion–related components (e.g., selenium) were associated with CVD mortality in summer and CVD hospitalizations in winter, whereas elemental carbon and NO_2_ showed associations with these outcomes in both seasons.

**Conclusion:**

Local combustion sources, including traffic and residual oil burning, may play a year-round role in the associations between air pollution and CVD outcomes, but transported aerosols may explain the seasonal variation in associations shown by PM_2.5_ mass.

Recent reviews of the health effects of particulate matter (PM) air pollution suggest evidence of adverse effects on cardiovascular disease (CVD) outcomes [e.g., U.S. Environmental Protection Agency (EPA) 2009]. Although several mechanisms such as oxidative stress and inflammation have been suggested (Brook et al. 2010; Mills et al. 2009), it is not clear if specific chemical constituents of PM play distinct roles in any of these mechanisms. If PM toxicity could be determined based on specific chemical constituents or source types that emit such constituents, the regulation of PM may be implemented more effectively. Also, if a given adverse health outcome is associated with specific PM component(s) but not with others, then a specific mechanism may be postulated for further consideration by toxicological studies.

Recent multicity time-series studies of PM have generated important information regarding the possible role of sources but have also raised some issues that require further investigation. For example, the most comprehensive U.S.-based multicity study of elderly hospital admissions (Bell et al. 2008) found the largest risk estimates for PM mass with aerodynamic diameter ≤ 2.5 μm (PM_2.5_; collected with a size-selective inlet with 50% cut-point of 2.5 μm aerodynamic diameter) for both cardiovascular and respiratory hospitalizations in northeastern cities and during the winter season. The most comprehensive U.S.-based multicity study (Peng et al. 2005) of all-cause mortality (of which nearly half are from cardiovascular causes) similarly found the largest PM_10_ (aerodynamic diameter ≤ 10 μm) risk estimates in the northeastern cities but during the summer season. Thus, although both the hospitalization and mortality multicity studies found the strongest associations in northeastern cities, their seasonal patterns of associations are not consistent.

Several studies have examined the annual or seasonal average levels of PM_2.5_ chemical components as potential effect modifiers in a second-stage regression in order to try to explain city-to-city variation in short-term PM risk estimates from the first-stage time-series analysis in individual cities. Lippmann et al. (2006) reported that some of the city-to-city variation in the PM_10_–mortality risk estimates from the National Morbidity, Mortality, and Air Pollution Study could be explained by the variation in nickel (Ni) and vanadium (V) contents in PM_2.5_ across cities. However, Dominici et al. (2007) pointed out, in their analysis, that such a finding could be highly influenced by New York City (NYC) data (NYC has a large population and high Ni levels). A recent analysis by Bell et al. (2009) reported that the city-to-city variations in PM_2.5_ risk for both cardiovascular and respiratory hospitalizations among elderly persons were significantly modified by Ni, V, and elemental carbon (EC), suggesting that PM_2.5_ effects were stronger in locations with higher residual oil-burning–related and traffic-related pollution, highlighting the importance of local combustion sources. Bell et al. (2009) also found that sulfate (SO_4_) was not a significant predictor of city-to-city variation in PM_2.5_ risks. In an analysis of 25 U.S. cities, which did not include NYC, Franklin et al. (2008) found that the PM_2.5_–mortality risk estimates were significantly modified by aluminum, arsenic, silicon (Si), SO_4_, and Ni. In their analysis of 27 U.S. cities, Zanobetti et al. (2009) observed that the association between PM_2.5_ and CVD hospitalizations was significantly modified when the PM was high in bromine (Br), chromium (Cr), Ni, and sodium ion (Na^+^). Overall, these studies show mixed results, and because PM_2.5_ chemical constituents were treated as effect modifiers of PM mass concentration effects on mortality or hospitalizations, they do not necessarily suggest that these individual chemical constituents are associated with the health outcomes on a day-to-day basis.

Few studies to date have examined daily PM chemical constituents in relation to health outcomes. In an analysis of Phoenix, Arizona, Mar et al. (2000) examined several key PM_2.5_ chemical components in addition to PM_2.5_ and gaseous pollutants. They found that EC was significantly associated with CVD mortality, but they also found similar associations with nitrogen dioxide (NO_2_), sulfur dioxide (SO_2_), carbon monoxide (CO), and PM_2.5_. Ostro et al.(2007) analyzed data from nine California counties and reported an association between cardiovascular mortality and several chemical components, including EC, organic carbon (OC), nitrate (NO_3_), iron, and potassium, at various lags. Peng et al. (2009) examined associations between elderly hospital admissions and seven PM_2.5_ chemical constituents that contributed substantive mass fractions to overall mass (EC, OC, SO_4_, NO_3_, Na^+^, Si, and ammonium ion) in 119 U.S. cities and found that EC was significantly associated with CVD hospitalizations. These studies either did not examine the PM_2.5_ chemical species whose mass contributions were low (e.g., Ni, V) or conduct analyses in the cities where the levels of these species were low.

The mortality and hospitalization studies noted above have examined the same or similar range of categories based on the *International Classification of Diseases, 9th Revision* [ICD-9; World Health Organization (WHO) 1977] and *10th Revision* (ICD-10; WHO 2007) for CVD, but none of them examined both CVD mortality and hospitalizations in the same analysis or compared how these two CVD outcomes relate to each other or to other covariates in the regression models. Investigation of the apparent discrepancy in seasonal and regional pattern of associations between PM and these outcomes would benefit from a direct comparison of these CVD outcomes and characterizations of their relationships to PM_2.5_ chemical constituents and other pollutants.

The objective of our analysis was to examine the role of key PM_2.5_ chemical constituents on both CVD hospitalizations and mortality in NYC. NYC has one of the nation’s highest levels of Ni, presumably from the combustion of relatively “dirty” residual oils in large buildings for space heating (Peltier et al. 2009), as well as from the ships in the Port of New York ship terminals in Newark and Elizabeth, New Jersey, that burn so-called “bunker fuels,” which are heavy residual oils. NYC also has the nation’s highest density of traffic. In addition, during warm seasons, a major fraction (50–80%) of PM_2.5_ in NYC is transported secondary SO_4_ and associated chemical constituents (Lall and Thurston 2006). NYC’s very large population also provided sufficient statistical power to examine the association between the CVD outcomes and PM_2.5_ chemical speciation data that were collected every third day.

## Materials and Methods

### Cardiovascular hospitalization and mortality data

Hospitalizations and mortality data for five boroughs (Manhattan, Brooklyn, Queens, the Bronx, and Staten Island) of NYC for the years 2000 through 2006 were available at the New York City Department of Health and Mental Hygiene (NYCDOHMH). The hospitalization discharge data were from the Statewide Planning and Research Cooperative System of New York State. The relevant variables available in the electronic discharge abstract for each patient included date of admission, age, sex, and primary ICD-9 discharge diagnosis code. The mortality records were obtained from the New York City Office of Vital Statistics. The following variables were used in our study: date of death, age, and the ICD-10 codes for underlying cause of death. We extracted hospitalization records whose type of admission was “emergency” or “urgent” only. For both hospitalizations and deaths, we aggregated daily counts for those ≥ 40 or more years of age for the following categories: hypertensive diseases (ICD-9, code 402; ICD-10, code I11); myocardial infarction (ICD-9, code 410; ICD-10, codes I21–I22); ischemic heart disease (ICD-9, code 414; ICD-10, code I25); dysrhythmias (ICD-9, code 427; ICD-10, code I48); heart failure (ICD-9, code 428; ICD-10, code I50); and stroke (ICD-9, codes 430–439; ICD-10, codes I60–I69). As expected, no mortality counts were found for dysrhythmias. We combined the daily counts for these specific causes for overall CVD mortality and overall CVD hospitalizations and used them for our main analysis but also examined the specific CVD series separately to check consistency of their results with the overall series.

### Air pollution and weather data

Data for PM_2.5_, NO_2_, CO, and SO_2_ were retrieved from the U.S. EPA Air Quality System (U.S. EPA 2008). Our previous study in NYC (Ito et al. 2007) found that PM_2.5_ and NO_2_ show generally high monitor-to-monitor temporal correlations, whereas CO and SO_2_ show generally poor monitor-to-monitor temporal correlations such that excluding certain monitors was not meaningful. Therefore, all available monitors within the five boroughs of NYC for these pollutants were included, and the average of data from multiple monitors was computed using the 24-hr average values. There were only three U.S. EPA PM_2.5_ chemical speciation monitors; our previous analysis of temporal correlations of PM_2.5_ chemical species at these three monitors (two in the Bronx and one in Queens, several miles apart) found that the extent of monitor-to-monitor correlation varied across chemical species, with higher correlations for the species associated with regional pollution and lower correlations for the species associated with local pollution (Ito et al. 2004). However, there was no clear justification for excluding certain monitors given the closeness of the monitors. Therefore, we also used the average of the three monitors’ data for this analysis. The sampling frequency of the chemical speciation data was every third day. This procedure resulted in a reduced sample size for PM components compared with other pollutants. The 24-hr average temperature and dew point for LaGuardia Airport were obtained from the National Oceanic and Atmospheric Administration, National Climatic Data Center (2009) Global Summary of the Day database.

More than 40 PM_2.5_ chemical species were available, but many of them had high percentages of observations below their detection limits or poor monitor-to-monitor correlations. Also, many species were of no interest in terms of source identification or suspected toxicity. Therefore, we selected the following key PM_2.5_ chemical components based on past source-apportionment studies in NYC (e.g., Ito et al. 2004; Lall and Thurston 2006; Qin et al. 2006) and recent health effects studies discussed in the introductory remarks: EC, OC, Ni, V, zinc (Zn), SO_4_, selenium (Se), Br, NO_3_, and Na^+^.

### Exploratory data analysis

We conducted several exploratory data analyses to describe and characterize the difference in temporal patterns between CVD hospitalizations and mortality and their relationships to weather and air pollution.

To characterize relative variance contributions from seasonal trends, day-of-week, and random components to the overall time series, we conducted spectral analysis of CVD hospitalizations and mortality. We used modified Daniel smoothers to smooth periodograms, applying several spans of smoothing over frequency, as suggested by Venables and Ripley (2002).

To characterize bivariate temporal relationships among weather, air pollution, and health outcome variables, we computed the cross-correlation function (CCF; correlation with lags) for key variables considered. These cross-correlations can indicate the sequence of temporal fluctuations (i.e., which variable leads the other in time). The correlation between two time series can be strongly influenced by shared trends, seasonal cycles, and day-of-week patterns that may be confounding. Therefore, in order to remove the influence of these temporal patterns and to focus on the short-term relationships between the variables, each time series was first prefiltered in a generalized linear model using a natural cubic spline smoothing function with 8 degrees of freedom per year and a day-of-week variable. A CCF was then computed using pairwise complete observations between the lagged residual time series. We chose 8 degrees of freedom per year to be consistent with the extent of adjustment for temporal trends in recent multicity time-series analyses (e.g., Bell et al. 2009; Samoli et al. 2008). Because relationships among these variables can change across seasons, the CCF was computed in a series of 12 three-month blocks centered on each month of the year and pooled for the entire 7-year study period.

### Regression models

We estimated percent excess risk (%ER) for air pollutants using a Poisson time-series model, adjusting for temporal trends and seasonal cycles, immediate and delayed temperature effects, and day of the week. The extent of lagged days considered for air pollution and weather variables were based mainly on recent time-series studies and the exploratory analysis of these data described above. We used natural cubic splines of days to adjust for potentially confounding temporal trends and seasonal cycles using 8 degrees of freedom per year in our base model. To adjust for immediate and delayed nonlinear temperature effects, our base model also included natural cubic splines of the same-day and the average of past 1- through 3-day lagged temperature with 3 degrees of freedom over the range for each term. Temperature and dew point were highly correlated (*r* = 0.93) in this data set, so we did not include them simultaneously. We did, however, compute apparent temperature from temperature and dew point (Steadman 1979) and included it in a separate alternative model for sensitivity analysis. Risks were estimated at lags 0 through 3 days for interquartile-range (IQR) increases of pollutants for all-year data (Table 1) and for all-year, warm (April through September), and cold (October through March) seasons. All the statistical analyses were conducted using the R statistical software package (version 2.10.1; R Development Core Team 2009).

## Results

**Figure 1:**
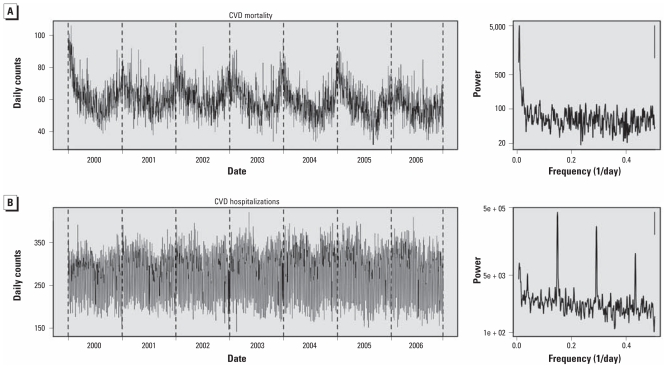
Time-series plots and power spectra of CVD mortality (*A*) and hospitalizations (*B*), with associated power spectra. Power spectra were estimated using modified Daniel smoothers over five and then seven frequencies; the bars in the upper right corner indicate 95% confidence bands of spectra.

CVD mortality and hospitalization time series exhibited markedly different characteristics. Figure 1 shows time series and corresponding power spectra of these time series. CVD mortality showed a strong seasonal pattern, with broad winter peaks that vary from year to year, apparently reflecting influenza epidemics. A day-of-week pattern was not apparent in the CVD mortality power spectra, which showed a major variance contribution in the frequency range corresponding to seasonal cycles. In contrast, CVD hospitalizations showed substantial variance contribution from day-of-week cycle frequencies (0.14/day or 7-day cycle, and its harmonics), which is stronger than that from seasonal cycles. CVD hospitalizations were lower on Saturday and Sunday, with hospitalizations about 60% higher on Monday than on Sunday. Likewise, the power spectra for specific CVD hospitalizations all showed strong day-of-week peaks that were stronger than those for seasonal cycles (data not shown).

Table 1 shows distributional characteristics of CVD outcome, weather, and air pollution variables. The largest fraction of CVD mortality was from ischemic heart disease, followed by myocardial infarction. CVD hospitalizations were more evenly divided by the specific categories. Ni, V, Zn, and SO_2_, all likely associated with residual oil burning, showed substantially higher levels in cold seasons than in warm seasons. Correlations among air pollutants by season are shown in Supplemental Material, Table 1 (doi:10.1289/ehp.1002667).

The following results from CCF and regression analysis necessarily involve multiple testing because of multiple lags, pollutants, and outcomes examined. Therefore, to guard against overinterpretation of chance findings, we focus not on the statistical significance of individual results but instead on identifying consistent patterns and contrasts of results across the lags, pollutants, and outcomes.

**Figure 2:**
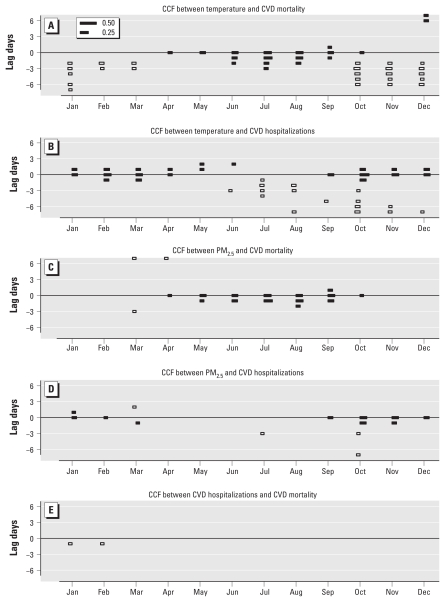
Cross-correlation function (CCF) between X = temperature, Y = CVD mortality (*A*); X = temperature, Y = CVD hospitalizations (*B*); X = PM_2.5_, Y = CVD mortality (*C*); X = PM_2.5_, Y = CVD hospitalizations (*D*); and X = CVD hospitalizations, Y = CVD mortality (*E*): a correlation below the centerline (lag 0) indicates that X leads Y, and a correlation above the centerline indicates that Y leads X. Black bars are positive correlations, and white bars are negative correlations (correlations whose absolute values are < 0.1 are not shown). The lengths of reference correlations (0.25 and 0.50) are also shown. CCFs were computed after removing seasonal trends and day-of-week pattern (see “Results”). Abbreviations: X, predictor; Y, outcome.

In the CCF analysis of key variables, after removing seasonal cycles and day-of-week patterns, temperature showed different patterns of associations with CVD mortality versus CVD hospitalizations, respectively (Figure 2A,B). With CVD mortality, temperature showed positive associations in summer and lagged negative associations in winter, consistent with fairly well-known mortality impacts of temperature reported in the past literature (e.g., Curriero et al. 2002). In contrast, CVD hospitalizations showed immediate and positive associations with temperature in the cold season, clearly not heat events, and biologically implausible. There was also a suggestion of lagged negative associations between temperature and CVD hospitalizations in the warm season and the beginning of the cold season. We repeated the analysis using apparent temperature rather than temperature (data not shown), but the pattern and magnitude of associations were essentially unchanged.

The CCF relationships between PM_2.5_ and CVD mortality and hospitalizations also showed contrasting patterns (Figure 2C,D). PM_2.5_ was positively associated with CVD mortality in the warm season with lags 0 and 1 day, whereas it was positively associated with CVD hospitalizations in the cold season, most consistently at lag 0 day. The CCF for chemical components of PM_2.5_ versus CVD outcomes showed less distinct patterns (data not shown), likely in part due to the smaller sample size (only every third day). The CCF relationship between temperature and PM_2.5_ chemical components showed generally positive associations on the same day throughout the year and delayed negative associations in cold seasons, but the relationship between temperature and Ni showed negative associations with longer lags and no positive associations in the cold season [Supplemental Material, Figure 1d (doi:10.1289/ehp.1002667)].

We found no correlation between CVD hospitalizations and CVD mortality, except for as weak negative correlation in the cold season (Figure 2E). This is consistent with the contrasting relationships between these CVD outcomes and temperature or PM_2.5_. The CCFs between specific CVD hospitalizations and corresponding CVD mortality (e.g., CCF between heart failure hospitalizations and heart failure mortality) likewise did not show associations (data not shown).

**Figure 3:**
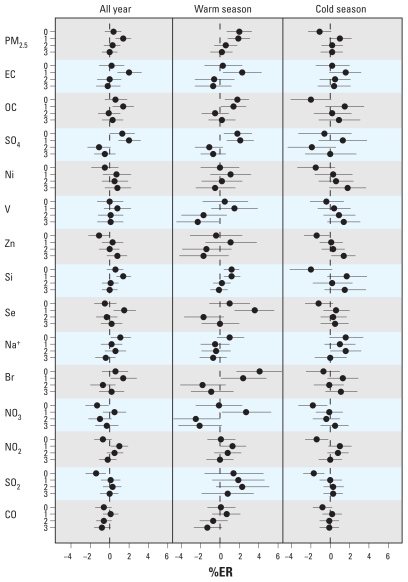
%ER for CVD mortality per IQR increase in air pollutant, adjusted for temporal trend, day-of-week, same-day, and delayed temperature effects.

Figure 3 shows air pollution–CVD mortality regression results (see Table 1 for IQRs used to compute %ER). PM_2.5_ was associated with CVD mortality in the warm season at lag 0 [%ER = 2.0%; 95% confidence interval (CI), 0.7–3.3; per 10 μg/m^3^] and lag 1 day (1.9%; 95% CI, 0.8–3.1) and in the cold season at lag 1 day (1.0%; 95% CI, −0.1 to 2.2). OC and SO_4_, both of which are associated with a major mass fraction of PM_2.5_ during the warm season (i.e., secondary aerosols), also showed significant associations with CVD mortality in the warm season at lags 0 and 1 days, with estimated %ERs similar to those for PM_2.5_ per IQR increase. Se, primarily associated with transported coal emissions in the eastern United States, and also associated with transported SO_4_, showed a strong association with CVD mortality at lag 1 day. EC was significantly associated with CVD mortality at lag 1 day in the warm season and was nearly significant in the cold season also at lag day 1. Br (perhaps associated with traffic) was strongly associated with CVD mortality at lag 0 in the warm season. Ni, V, and Zn (the elements most associated with residual oil burning in NYC) showed a similar pattern of associations with CVD mortality, with stronger associations in the cold season at lag 3 days. Na^+^ (considered to be a signature element for sea salt) also showed nearly a significant positive association at lag 0 day in both warm and cold seasons. Among the gaseous pollutants, NO_2_ showed lag 1-day positive associations with CVD mortality in both warm and cold seasons. There are peculiar negative (significant or nearly significant) associations for a number of pollutants at lag 0 day in the cold season (PM_2.5_, OC, Ni, Zn, Si, Se, NO_3_, NO_2_, SO_2_, and CO). These pollutants were not negatively correlated with CVD mortality in the CCF analysis (data not shown), and the negative associations may be induced by the adjustment for temperature. Not surprisingly, among the specific CVD mortality categories, mortality for ischemic heart disease showed a similar pattern of association as that for total CVD mortality (data not shown).

**Figure 4:**
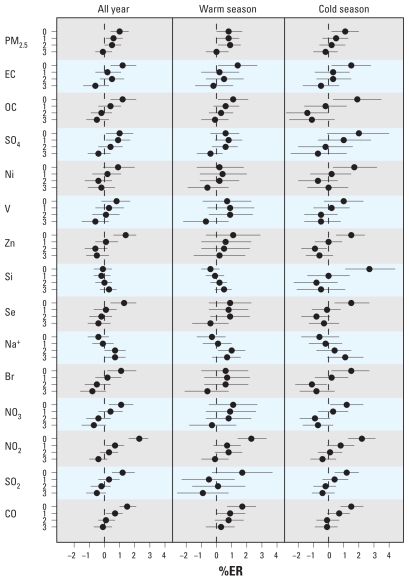
%ER for CVD hospitalizations per IQR increase in air pollutant, adjusted for temporal trend, day-of-week, same-day, and delayed temperature effects.

The relationships between air pollutants and CVD hospitalizations showed a pattern of association different than that for CVD mortality (Figure 4). The associations between air pollution and CVD hospitalizations, in contrast to CVD mortality, were generally stronger in the cold season than in the warm season. The exceptions were EC, NO_2_, SO_2_, and CO, which showed similar magnitude and lag structure of associations in both seasons. PM_2.5_ exhibited the strongest association at lag 0 day in the cold season (%ER = 1.1; 95% CI, 0.2–2.0), and most of the PM_2.5_ chemical components that showed significant or near significant associations (OC, SO_4_, Ni, Zn, Si, Se, and Br) with CVD hospitalizations showed the strongest associations at lag 0 day in the cold season. Of the specific CVD hospitalizations, those for hypertensive disease, ischemic heart disease, dysrhythmias, and stroke showed patterns of associations similar to that for total CVD hospitalizations (data not shown). Seasonal patterns of associations, lags, source types, and %ER are summarized for CVD mortality and hospitalizations in Supplemental Material, Table 2 (doi:10.1289/ehp.1002667).

These results were not sensitive to the alternative model in which we used apparent temperature, rather than temperature, to adjust for weather effects. Supplemental Material, Figure 2 (doi:10.1289/ehp.1002667), shows comparisons of risk estimates for CVD mortality and hospitalizations using temperature and apparent temperature for the warm and cold seasons. There appears to be no systematic difference in the risk estimates obtained from these models.

## Discussion

We found that adult CVD mortality and emergency CVD hospitalizations time series, when constructed with consistent ICD categories, were not correlated over time. The CVD mortality series showed a strong influence of seasonal cycles and influenza, whereas CVD hospitalizations were strongly influenced by a day-of-week pattern, with little seasonal pattern. Their relationships with temperature, as examined in our seasonal CCF analysis, were also different. The strong influence of the day-of-week pattern seen in CVD hospitalizations (lower counts on weekends) may imply that they are more strongly influenced by behavioral modification factors than by CVD mortality (e.g., being able to delay seeking treatment over the weekend). Also, it is possible that coding practice and conditions under which a “primary cause” is assigned to these outcomes may be different. For example, if a person with a chronic ischemic heart condition dies with an additional complication of influenza-related pneumonia, the primary cause of death may still be recorded as a heart-disease–related cause, whereas when a person with an acute cardiovascular condition is admitted to a hospital through the emergency department, the primary cause may be less ambiguous. If such differences contribute to the apparent lack of correlation between these two CVD outcomes, then the subgroups at risk for PM effects within these CVD outcomes need not share the same risk characteristics or even the same mechanism(s) of PM effects. However, in this analysis, we did not specifically examine whether some of those who were hospitalized died later, or whether a subgroup’s hospitalizations were associated with air pollution. If the fraction of each subgroup is relatively small in the overall CVD hospitalizations, then we may not see significant lagged correlation between CVD hospitalizations and mortality, whether or not they are associated with air pollution in the same causal pathway.

CVD mortality’s immediate association with high temperature in the warm season and lagged association with cold temperature in the cold season found in our analysis are consistent with expectation and the past literature. However, the lack of heat effects on CVD hospitalizations in warm seasons and the presence of positive associations in cold seasons (i.e., the warmer the temperature, the more CVD hospitalizations) found in this study is somewhat surprising, although it is in part consistent with “a protective effect of cold temperature” (i.e., the lack of the left side of the U-shape in the temperature–outcome relationship) that was reported by Schwartz et al. (2004) who analyzed temperature and elderly CVD hospitalizations in 12 U.S. cities. Also, in another analysis of CVD hospitalizations in NYC, whereas the overall CVD hospitalizations were positively associated with high temperature, the hospitalizations for CVD, heart failure, and hypertension showed negative risk estimates (Lin et al. 2009). Thus, there may be heterogeneity in temperature effects within subcategories of CVD hospitalizations.

In these analyses, CVD mortality was more strongly associated with PM_2.5_ in the warm season than in the cold season, whereas CVD hospitalization was more strongly associated with PM_2.5_ in the cold season than in the warm season. These findings are consistent with the results of the analysis in the U.S. Northeast of (all-cause) mortality and PM_10_ (Peng et al. 2005) and the analysis of CVD hospitalizations and PM_2.5_ (Bell et al. 2008). The lag structures of PM_2.5_ associations for CVD mortality (lags 0 and 1 day) and CVD hospitalizations (lag 0 day) are consistent with those found in past multicity studies for mortality (Peng et al. 2005) and CVD hospitalizations (Bell et al. 2008; Dominici et al. 2006; Peng et al. 2009).

The patterns of lagged associations between individual PM_2.5_ chemical components and CVD mortality were not always consistent with those for PM_2.5_ mass concentrations. It is not surprising that SO_4_ and OC were associated with CVD mortality on the same lag days (0 and 1) as PM_2.5_ because both SO_4_ and OC contribute major mass fractions to PM_2.5_. Si, a marker of soil whose associated species together contribute significant amounts of particle mass, also showed the same lag structure of associations. However, other PM chemical components with smaller mass contributions showed different lagged associations with CVD mortality compared with PM_2.5_. Ni, V, and Zn (i.e., the species associated with residual oil burning in NYC) all showed delayed associations (largest estimates at lag 3 days) with CVD mortality. However, this may reflect the relationship between temperature and these species in the cold season [see Supplemental Material, Figure 1 (doi:10.1289/ehp.1002667)], which was negative with long lags and had no positive associations on the same day. Therefore, caution is required in interpreting these lagged associations between PM components and health outcomes, because they may have varying relationships with other covariates in the regression model. Also, the 3-day lagged association between Ni and CVD mortality is not necessarily consistent with Ni’s effect modification of 1-day lagged PM_10_–mortality associations suggested in Lippmann et al.’s (2006) result.

We observed the associations between CVD hospitalizations with air pollutants primarily in the cold season, except for EC, Na^+^, NO_2_, SO_2_, and CO, which were associated in both seasons. Because most of the PM_2.5_ chemical components and gaseous pollutants examined similarly yielded significant or nearly significant associations at lag 0 day, it is difficult to draw inferences on which chemical components or sources may be more important for the PM_2.5_ association with CVD hospitalizations in this data set. These results are, in part, consistent with the significant effect modifiers (EC and Ni) of PM_2.5_ effects on elderly CVD hospitalizations reported in the Bell et al. (2009) analysis or those (Br and Ni) reported in the Zanobetti et al. (2009) analysis but show a broader spectrum of PM_2.5_ source components contributing to effects.

Given the lack of correlation between CVD hospitalizations and CVD mortality over time, we did not expect “coherence” in specific PM_2.5_ chemical components’ association with these two CVD outcomes. Of the pollutants examined, EC and NO_2_ showed the most consistent associations with these CVD outcomes, in that these pollutants showed the same extent of associations in both warm and cold seasons with the same lags (lag 1 day for CVD mortality and lag 0 day for CVD hospitalizations). Both EC and NO_2_ are often considered to be “signature” tracers for traffic sources. However, EC may be emitted from other fuel combustions, including oil boiler emissions (Schauer 2003). Likewise, NO_2_ is also emitted from a variety of combustion sources. In fact, in the New York City Community Air Survey conducted during the winter of 2008 and 2009, which measured PM_2.5_, its chemical components, and gaseous pollutants at 150 locations in NYC, land-use regression analysis found that both traffic and building density (i.e., proxy for building space heating) were significant predictors of EC and NO_2_ in this city (NYCDOHMH 2009). This does not directly imply that these pollutants per se are responsible for the health effects, but they may be important indicators of sources relevant to the PM_2.5_ effects on CVD outcomes.

The relative significance of the associations of the PM components with CVD outcomes could have been influenced by their corresponding exposure misclassification errors that include the error associated with the locations of monitors relative to the population. An investigation related to this issue involving the same three speciation monitors (Ito et al. 2004) found that the PM_2.5_ chemical species associated with regional secondary aerosols (e.g., SO_4_ and NO_3_) tend to have higher monitor-to-monitor temporal correlations than do the species associated with more local combustion sources (e.g., EC and Ni). Thus, the interpretation of our study findings is limited by the uncertainty regarding the influence of relative exposure error across the species on the observed associations.

## Conclusion

We found that CVD hospitalizations and CVD mortality time series are not correlated, but they are each independently associated with a number of PM_2.5_ chemical components, including both regional secondary aerosols and local combustion sources. EC and NO_2_ showed the most consistent associations with these CVD outcomes throughout the year, but the PM components associated with the regional transported aerosols from coal combustion (Se and SO_4_) exhibited the season-specific association pattern most similar to that of PM_2.5_ mass. We conclude that local combustion sources, including traffic and residual oil burning, may be important sources affecting CVD adverse effects in NYC.

## Figures and Tables

**Table 1:** Distribution of daily CVD mortality and hospitalization, weather, and air pollution variables.

	All year		Warm season	Cold season
Variable	Mean ± SD	IQR^a^	Mean ± SD	Mean ± SD
Mortality (counts/day)				

Hypertension	3.5 ± 2.0	3.0	3.3 ± 1.9	3.6 ± 2.0
Myocardial infarction	10.7 ± 3.8	5.0	9.9 ± 3.7	11.5 ± 3.8
Ischemic heart disease	42.1 ± 8.2	11.0	39.4 ± 7.1	44.8 ± 8.4
Heart failure	1.7 ± 1.4	2.0	1.7 ± 1.3	1.8 ± 1.4
Stroke	1.8 ± 1.4	2.0	1.8 ± 1.4	1.9 ± 1.4
CVD (sum of above)	59.8 ± 10.4	13.0	56.0 ± 8.7	63.7 ± 10.6

Hospitalizations (counts/day)				

Hypertension	5.5 ± 2.9	4.0	5.3 ± 2.9	5.6 ± 2.9
Myocardial infarction	41.0 ± 9.0	12.0	39.6 ± 8.5	42.4 ± 9.3
Ischemic heart disease	64.6 ± 25.2	47.0	64.9 ± 24.9	64.3 ± 25.5
Dysrhythmia	41.0 ± 11.1	17.0	41.6 ± 11.2	40.4 ± 11.0
Heart failure	74.8 ± 16.5	24.0	72.3 ± 16.2	77.4 ± 16.4
Stroke	54.4 ± 9.7	13.0	54.8 ± 9.4	54.0 ± 10.0
CVD (sum of above)	281.3 ± 58.8	106.0	278.6 ± 58.1	284.0 ± 59.5

Weather (°C)				

Temperature	13.2 ± 9.49	16.04	20.22 ± 6.08	6.16 ± 6.67
Dew point	5.81 ± 10.18	16.57	12.78 ± 6.8	−1.18 ± 7.97
Apparent temperature	12.6 ± 10.96	19.32	20.73 ± 8.04	4.44 ± 6.56

PM_2.5_/components (μg/m^3^)				

PM_2.5_	14.44 ± 8.53	10.0	14.79 ± 8.99	14.09 ± 8.02
EC	1.13 ± 0.56	0.62	1.03 ± 0.51	1.24 ± 0.58
OC	4.3 ± 2.28	2.38	4.51 ± 2.62	4.08 ± 1.83
SO_4_	4.14 ± 3.29	3.19	4.81 ± 4.01	3.42 ± 2.04
NO_3_	2.12 ± 1.98	2.19	1.52 ± 1.32	2.78 ± 2.33
Na^+^	0.14 ± 0.11	0.11	0.14 ± 0.1	0.15 ± 0.11
Ni	0.0171 ± 0.0133	0.0144	0.0111 ± 0.0097	0.0236 ± 0.0136
V	0.0066 ± 0.0045	0.0053	0.0054 ± 0.0035	0.0080 ± 0.0050
Zn	0.0300 ± 0.0208	0.0194	0.0216 ± 0.0116	0.0389 ± 0.0245
Si	0.0769 ± 0.0712	0.0543	0.0886 ± 0.0898	0.0643 ± 0.0394
Se	0.0013 ± 0.0012	0.0013	0.0011 ± 0.0010	0.0015 ± 0.0014
Br	0.0036 ± 0.0025	0.0031	0.0031 ± 0.0020	0.0040 ± 0.0028

Gaseous pollutants				

NO_2_ (ppb)	28.7 ± 8.8	11.5	27.5 ± 8.6	30.0 ± 8.9
SO_2_ (ppb)	7.4 ± 6.1	6.6	3.9 ± 2.6	10.8 ± 6.7
CO (ppm)	1.0 ± 0.43	0.5	0.94 ± 0.35	1.06 ± 0.49

a IQRs for pollution variables on this table were used for the risk calculation.
